# The effectiveness of transcranial electrical stimulation in individuals with specific learning disorder (SLD): systematic review and transfer analysis

**DOI:** 10.1186/s11689-025-09623-7

**Published:** 2025-11-05

**Authors:** Vahid Nejati, Fateme Ghafuri, Katayoon Hosseini, Roozbeh Behroozmand

**Affiliations:** 1 School of Brain and Behavioral Sciences, University of Texas at Dallas, Dallas, USA; 2 Department of Psychology, Shahid Beheshti University, Tehran, Iran; 3 Department of Psychology, University of Tabriz, Tabriz, Iran

**Keywords:** Specific learning disabilities (SLD), Transferability, Transcranial electrical stimulation (tES), Review, Meta-analysis, Effectiveness

## Abstract

This comprehensive review aimed to investigate the transferability of transcranial electrical Stimulation (tES) interventions in individuals with specific learning disabilities (SLD) based on the FIELD model, encompassing function, implements, ecology, level, and durability. A systematic search of electronic databases yielded a total of 13 eligible studies, 11 transcranial direct current stimulation (tDCS), 1 transcranial random noise stimulation (tRNS), and 1 transcranial alternating current stimulation (tACS), encompass 286 individuals with SLD, for inclusion. The overall effect size analysis revealed positive transfer effects in all domains of FIELD, indicating the potential effectiveness of non-invasive brain stimulations (NIBS) interventions in enhancing various aspects of learning and behavior in individuals with SLD. The subgroup analysis further underscored the positive impact of age, dose, and concurrent intervention on transferability. In conclusion, this study contributes valuable insights into the transferability of tES interventions and holds promise for improving learning and behavioral outcomes in individuals with SLD.

## Introduction

Specific Learning Disabilities (SLD) encompass a diverse range of neurobehavioral conditions characterized by significant and persistent challenges in acquiring and utilizing efficient reading (dyslexia), writing (dysgraphia), or mathematical (dyscalculia) abilities, despite receiving conventional instruction, intact sensory and motor functioning, normal intelligence, proper motivation, and adequate sociocultural opportunities [3]. Indeed, SLD refers to abnormal cognitive processing including visual perception [14], auditory perception [52], spatial abilities [74], inhibitory control [69], and working memory [40, 65].

At the neural level, hypoactivation in the left inferior frontal cortex (IFC) and the anterior cingulate cortex (ACC), as well as the left occipito-temporo-parietal regions, has been described during writing in dyslexia [38]. In contrast, hyperactivation has been observed in the right postcentral gyrus, the left rectus, and the right middle temporal gyrus in individuals with dyslexia [38]. Similarly, hyperactivity [20, 58] or hypoactivity [4, 7] of the frontoparietal network has been described in individuals with dyscalculia. Furthermore, a decrease in brain activity has been observed in the prefrontal cortex [4, 7] as well as the posterior parietal region, including the inferior parietal sulcus [4, 7, 21], as reported in earlier studies.

In addition to the aforementioned neuroimaging studies with correlational findings, non-invasive brain stimulations (NIBS) offer valuable causal evidence regarding the neural underpinnings of impaired cognitive functions and educational skills in individuals with SLD. There are two main NIBS techniques: transcranial electrical stimulation (tES) and transcranial magnetic stimulation (TMS). tES involves applying an electrical current to the brain, which leads to changes in cortical excitability [48]. Among tES methods, three stand out: transcranial direct current stimulation (tDCS), transcranial alternating current stimulation (tACS), and transcranial random noise stimulation (tRNS). Specifically, tDCS is known for its ability to modify neuronal resting membrane potentials and, depending on the stimulation polarity, either enhance or reduce the excitability of the targeted cortical area at a macroscopic level [47]. In tACS, an alternating current is applied over the scalp to entrain or synchronize cortical oscillations [2]. On the other hand, tRNS involves applying an alternating current with distributed frequencies to stimulate different types of neurons and promote desynchronization of various cortical rhythms [50]. As for TMS, it induces a magnetic field in the target regions of the brain, leading to suprathreshold depolarization of the targeted cortical neurons [72]. Depending on the frequency of stimulation, TMS can have an inhibitory effect with low-frequency stimulation (< 1 Hz) or an excitatory effect with high-frequency stimulation (> 5 Hz). For experimental purposes, TMS can deliver monophasic pulses in single-pulse experiments or biphasic pulses in repetitive TMS experiments [36].

For instance, in developmental dyslexia, several NIBS studies have shown promising effects on various cognitive aspects and reading abilities. Left anodal/right cathodal stimulation of the TPJ was found to enhance text accuracy, word recognition speed, motion perception, and modified attentional focusing [34, 35], as well as pseudoword reading in individuals with dyslexics [42], and to decrease errors in text reading [17, 18]. Additionally, anodal stimulation of the left superior temporal gyrus, with a right-side or extracranial return electrode, resulted in improved reading accuracy and speed [55]. Additionally, bilateral tRNS over the primary auditory cortex increases acuity in phoneme categorization [59]. Moreover, 30-Hz tACS over the left auditory cortex improves phonological processing and reading accuracy [41], while bilateral 40 Hz-tACS targeting the left auditory cortex increases phoneme categorization skills [59]. Lastly, hf-rTMS over the left IPL improves non-word reading accuracy, and hf-rTMS stimulation over the left STG increases word reading speed and text reading accuracy [15]. In the realm of dyscalculia, Rahimi et al. [54] demonstrated that anodal stimulation of the left superior temporal gyrus, combined with a right-side or extracranial return electrode, improved auditory comprehension and central auditory processing [54]. A previous comprehensive review study indicated that the application of repeated reading training sessions in conjunction with various NIBS protocols holds the potential to bring about enduring enhancements in reading performance among individuals with dyslexia, spanning both children and adults [61].

Moreover, NIBS techniques present an opportunity to potentially enhance abnormal brain functioning by modulating respective brain areas [11, 28, 48]. Several multisession studies described improved neural [49, 60], cognitive [6, 49], and behavioral [6, 16–18, 22, 26, 33–35, 39, 43, 49, 57, 60, 68] problems children with SLD.

As discussed previously, SLD is a multifaceted behavioral problem involving cognitive impairments and underlying neural abnormalities. The connection between dysfunctions at the neural, cognitive, and behavioral levels is causal rather than purely correlational. Consequently, interventions can be directed at each of these levels to address and manage SLD effectively. Transferability refers to the extent to which training effects can be spread from a trained domain to untrained domain(s) [25]. Transferability is often regarded as a crucial measure of intervention effectiveness, indicating whether an effective intervention can target the core pathology and lead to improvements that extend to all affected domains. In the context of the FIELD model, transfer encompasses five essential dimensions: Function, Implement, Ecology, Level, and Durability [46]. "Function" pertains to the capability of transferring performance from a trained function, like imitation, to an untrained function, such as theory of mind. The "Implement" dimension deals with the various materials and methods employed in the assessment and training process. Demonstrating improvement through diverse assessment tools, as opposed to intervention materials and methods, indicates the occurrence of implement transfer. "Ecological transfer" involves transferring intervention effects from a specific setting, such as a clinical environment, to an entirely different and new setting, like the home. "Level transfer" concentrates on transferring training effects across various levels, encompassing neural, cognitive, and behavioral levels. Lastly, "Durability" examines how long the effects of training persist after discharge, as illustrated in Table 1.

**Table 1:** Description of different transfer domains based on FIELD model

Dimension	Description
Function	The transfer of training effect from trained function to an untrained function(s)
Implement	Improvement demonstrated through diverse assessment tools during the assessment and training process
Ecology	The transference of intervention effects from one specific setting (e.g., clinic) to an entirely different and new setting (e.g. home)
Level	The transfer of training effects across different levels, including neural, cognitive, and behavioral levels
Duration	Concerned with how long the effects of training persist after discharge

This study aims to systematically review previous findings on NIBS techniques as interventions for SLD, focusing on their cognitive and neural effects across different domains. In addition, we conducted a meta-analysis to quantitatively assess the impact of these interventions specifically within the framework of the FIELD model, examining transferability. By combining a systematic review with a meta-analysis, we aim to both provide a comprehensive overview of the existing literature and quantitatively evaluate the extent to which NIBS can address core deficits in SLD across distinct FIELD dimensions.

## Method

The protocol for this systematic review has been registered on the PROSPERO website (www.crd.york.ac.uk/PROSPERO) with a unique registration number (CRD42023393408). The development of this systematic review protocol adheres to the guidelines set forth by the Preferred Reporting Items for Systematic Reviews and Meta-Analyses (PRISMA) statement. The following section details the study selection criteria, search strategy, data extraction, and risk of bias assessment for the systematic review, along with transfer measurement and analysis procedures for the meta-analysis.

### Study selection criteria

The systematic review encompassed all clinical trials with either parallel or cross-over design, without imposing restrictions on the number of arms. The studies considered were open-label (single, double, or triple blind) and could be either randomized or non-randomized. The outcome measures focused on any changes observed in academic performance (reading, writing, mathematics), cognitive effects, or neural effects.

This review encompassed all studies involving individuals with SLD, without imposing limitations on age, gender, or nationality of the participants. SLD considered in this review include SLD with impairment in reading or dyslexia, SLD with impairment in written expression, and SLD with impairment in mathematics or dyscalculia. Two reviewers (FG, KH) assessed the titles and abstracts of all primary articles identified through the search strategy to determine the studies eligible for inclusion. Subsequently, these two reviewers independently evaluated the full text of potentially relevant, non-duplicated articles (FG, KH). Any disagreements were resolved through discussion to achieve consensus. In cases where consensus was not achieved, a third reviewer (VN) served as an arbitrator to settle the matter.

### Search strategy

The following electronic databases were searched from 1990 through September 2024, with no restrictions on the English language: PubMed, Scopus, Web of Science, Eric (via EBSCO), and APA PsycINFO. Additionally, ongoing trials were sought in the Cochrane Central Register of Controlled Trials (CENTRAL), clinical trial registries including ClinicalTrials.gov and International Standard Randomized Controlled Trial Number (ISRCT), and the WHO network registry for trials. Relevant Grey Literature within our scope was identified by searching ProQuest for associated dissertations, and Web of Science and Scopus for conference papers. The search terms for this systematic review were extracted from Medical Subject Headings (MeSH), a standardized vocabulary used to index articles for PubMed and other databases, and the keywords of the studies in the primary search, based on the intervention and outcome components.

The search terms were adapted according to the specific database. The full search strategy for each database can be found in the supplementary materials. Additionally, bibliographies of all relevant prior reviews and primary studies identified by the search strategy were scanned for additional relevant papers.

### Data extraction

**Fig. 1:**
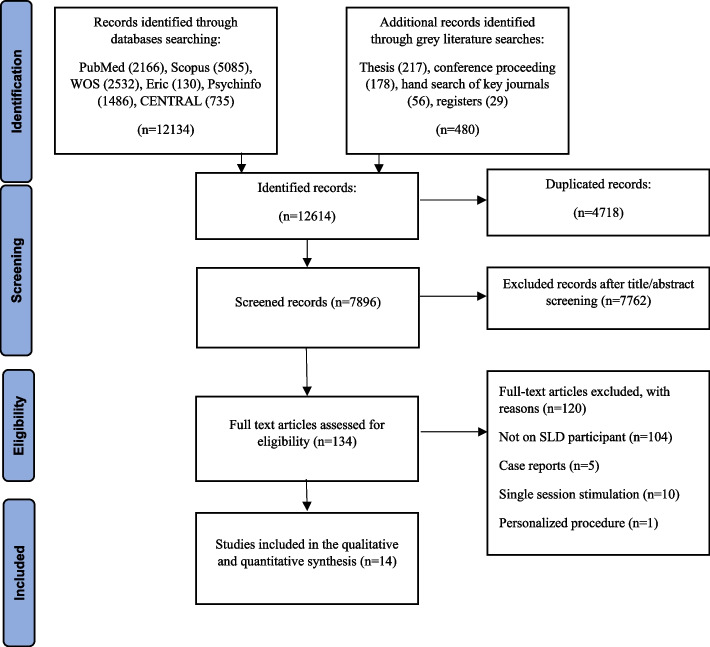
Data extraction diagram for review. 14 studies were entered into our study out of 12,614 initial candidate studies. 7762 studies were excluded in the screening phase, and 134 studies in the eligibility phase

Data extraction from primary articles was performed independently by two reviewers (FG, KH) using a data extraction form. Any discrepancies were resolved by consensus between the two reviewers and, when this was not possible, a third reviewer (VN) acted as an arbitrator. The extracted data included the name of the first author, year of publication of the article, the country of study, study design, type of intervention, sample size and participants'characteristics such as gender, age, and type of SLD, blinding, NIBS protocol (type of stimulation, electrodes size, intensity and frequency, duration, number of sessions), conditions with electrodes site, and behavioral or cognitive or neural outcome. Corresponding authors of included studies, in which required data were not provided, were contacted to request the data needed for the purpose of meta-analysis (Fig. 1).

### Risk of bias (quality) assessment

Risk of bias assessments were conducted using the Cochrane extension tool (https://www.cochrane.org). All studies were categorized into three groups based on their risk of bias: low risk, high risk, and unclear risk of bias. Accordingly, studies were assigned one of three grades: low, high, or unclear risk of bias. A low risk of bias indicates that the study is well-designed, with strong controls across each domain. A high risk of bias is given to studies with significant methodological issues, suggesting that the results may be affected by specific biases. An unclear risk of bias is used when there is insufficient information to determine the level of bias, often due to poor reporting or vague methods, making it difficult to assess the study’s reliability [13]. This grading system is visually represented with colors or symbols (e.g., "+", "-", "?") in Table 2. Two investigators (FG, KH) independently rated each study, and any discrepancies were resolved through consensus or by referring to a third investigator (VN). All studies were categorized into three groups based on their risk of bias: low risk, high risk, and unclear risk of bias.

**Table 2:**
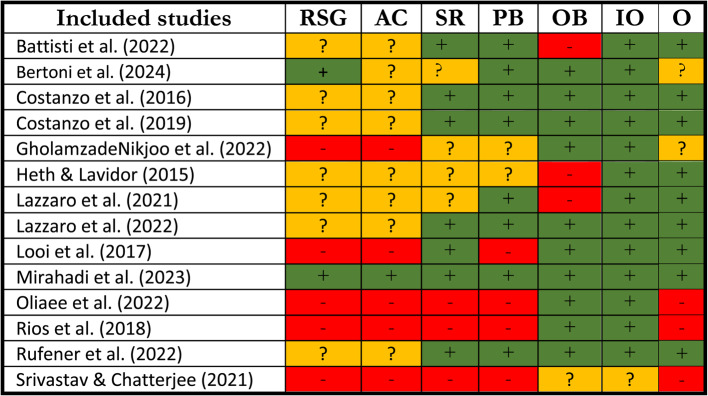
Risk of bias of included studies. Battisti et al. [6], Bertoni et al. [8], Costanzo et al. [16], Costanzo et al. [17, 18], GholamzadeNikjoo et al. [22], Heth & Lavidor [26], Lazzaro et al. [33], Lazzaro et al. [34, 35], Looi et al. [39], Mirahadi et al. [43], Oliaee et al. [49], Rios et al. [57], Rufener et al. [60], Srivastav & Chatterjee [68]

*Abbreviations*: *RSG* random sequence generation, *AC* allocation concealment, *SR* selective reporting, *PB* participants blinding, *OB* outcome blinding, *IO* incomplete outcome data, *O* other biases

color coding; green/+: low risk, yellow/?: unclear, red/-: high risk

### Transfer measurement and analysis

Based on the FIELD model of transfer, each assessment test is assigned a value within this framework. We calculate the effect size of the tests used in the studies and consider this effect size in relation to the corresponding domains of FIELD [46]. For example, considering a tDCS study that uses the N-back test, the N-back test evaluates working memory as its Function, is implemented through a computer as its Implement, operates at the Level of cognition, and is conducted in a clinical Environment during pretest, posttest, and follow-up sessions as the Duration of use.

Similarly, tDCS stimulation targets the electrical activity of the brain as its Function, utilizes a device as its Implement, occurs in a clinical Environment, and focuses on neural activity at the Level of intervention for the Duration of the pretest and posttest.

Mapping the tests and interventions reveals that similarities between the intervention and the test within each FIELD domain diminish the potential for transferability, while differences indicate the possibility of transfer. The respective effect size is then considered a measure of transfer in that domain, Table 3.

**Table 3:** An example of exploring transfer potential in a tDCS study using the n-back test

FIELD Domains	Assessment (N-back)	Intervention (tDCS)	Domain Similarity	Discovering Transferability
Function	Cognitive function (working memory)	Electrical activity	Different	Yes
Implement	Computer	Device/Stimulator	Different	Yes
Ecology	Clinic	Clinic	Similar	No
Level	Cognitive	Neural	Different	Yes
Duration	from pretest to follow-up	from pretest to post-test	Different	Yes

To quantify transfer within each FIELD domain, we derived values by summing the effect sizes of the relevant assessments. Table 3 presents the transfer FIELD values for both assessments and interventions across all studies, while the effect sizes of the corresponding tests are documented in Table 8 in Appendix for reference.

To measure side effects for different studies, different formulas were used. In certain situations, conducting an analysis based on changes from baseline can be more efficient and powerful compared to comparing final values. This approach eliminates a portion of between-person variability from the analysis, leading to more accurate and robust results. By focusing on changes within individuals over time, the impact of individual differences is minimized, allowing for a more sensitive detection of treatment effects or interventions. As a result, analyzing changes from baseline can provide a clearer picture of the true treatment effect and enhance the statistical power of the study [19]. To calculate the mean change in each group, we subtract the post-intervention mean from the baseline mean. A mean change for experimental group (E) was calculated using formula 1.

1 $${M}_{E.change}=\left({M}_{E. T2}-{M}_{E.T1}\right)$$

This represents the difference between the post-intervention score (T2) and the pretest or baseline score (T1). We computed the mean change for the control group (C) similarly, Formula 2.

2 $${M}_{C.change}=\left({M}_{C. T2}-{M}_{C.T1}\right)$$

The standard deviation of the change (SD_change_) from baseline for both the experimental and control groups was calculated using Formula 3.

3 $$\begin{array}{c}{SD}_{E.change}=\sqrt{{SD}_{E.T1}^{2}+{SD}_{E.T2}^{2}-(2*Corr\times {SD}_{E.T1}\times {SD}_{E.T2}})\\ { SD}_{C.change}=\sqrt{{SD}_{C.T1}^{2}+{SD}_{C.T2}^{2}-(2*Corr\times {SD}_{C.T1}\times {SD}_{C.T2}})\end{array}$$

Finally, effect sizes based on pre-post gains for the between-subject design were calculated using Formula 4.

4 $$d=\frac{\left({M}_{E. change}-{M}_{C.change}\right)}{\sqrt{\frac{{(n}_{E.T1}-1)\times {SD}_{E.change}^{2}+{(n}_{C.T1}-1)\times {SD}_{C.change}^{2}}{{n}_{E.T1}+{n}_{C.T1}-2}}}$$

In the nine included studies [6, 16–18, 22, 26, 34, 35, 43, 60], effect sizes based on pre-post gains for between-subject designs were calculated using Formula 5.

5 $$d=\frac{\left({M}_{E. change}-{M}_{C.change}\right)}{\sqrt{\frac{{(n}_{E.T1}-1)\times {SD}_{E.change}^{2}+{(n}_{C.T1}-1)\times {SD}_{C.change}^{2}}{{n}_{E.T1}+{n}_{C.T1}-2}}}$$

In some other studies [49, 57, 68], which reported pre-post comparisons for the experimental condition, the effect sizes were calculated using Formula 6.

6 $$d=\frac{\left({M}_{E. T2}-{M}_{E.T1}\right)}{{SD}_{E.T1}^{2}}$$

The effect size measures were transformed to Cohen's d effect size using the online platform psychometrica [37] for two studies [33, 39]. The effect sizes in different studies across various field domains were compared using Stata (Version 14.2) to weight the effect sizes based on sample size and the number of tests in each study. By incorporating sample size and the number of tests, the variability and reliability of effect size estimates across studies were taken into account. Weighting the effect sizes allowed giving more prominence to studies with larger sample sizes and more robust findings, while studies with smaller sample sizes or potentially less reliable results were down weighted. Stata was used to perform the transfer analysis procedures that considered the study-specific weights and generated pooled effect sizes that provided a more accurate representation of the overall effect across the field domains. This approach allowed for a comprehensive comparison of effect sizes from different studies and enabled the assessment of the combined effect more effectively. In this study, the effect sizes were interpreted using Hedges'g (Standardized Mean Difference for small sample sizes), and the benchmarks for categorizing the effect sizes were as follows: a small effect size was considered to be below 0.5, a medium effect size was 0.51–0.79, and a large effect size was 0.8 and upper. With these benchmarks, a consistent and meaningful comparison of effect sizes across different studies was allowed, enabling the evaluation of the practical significance and impact of the observed effects. By employing Hedges'g and these standardized benchmarks, valuable insights into the effectiveness and relevance of the interventions or treatments under investigation were gained, and the overall interpretability of the results was enhanced. Heterogeneity among the included studies was assessed using the I^2^ statistic, which quantifies the percentage of total variation in effect sizes attributable to heterogeneity rather than chance, with a value of I^2^ = 89.2% indicating a high level of heterogeneity. For the test of heterogeneity in the meta-analysis, we set the threshold for statistical significance at *p* < 0.05.

## Results

This review included a total of 14 studies with a participant pool of 306 individuals diagnosed with SLD. In the included studies, 11 utilized tDCS, while 2 applied tRNS, and 1 study employed tACS. Methodologically, 11 studies followed a randomized controlled trial (RCT) design, whereas 3 adopted a single-group pretest–posttest design. The participants consisted of 121 children, 146 adolescents, and 39 adults. The gender distribution included 116 females, 154 males, and 36 cases where gender was not specified. The subtypes of SLD identified in the study were 281 participants classified as dyslexia and 25 as dyscalculia subtypes. The diagnosis of SLD was based on DSM-5 (*n* = 10), and 4 studies not specifying the criteria. Nine out of 12 studies delivered an additional intervention, including cognitive training (*n* = 1), reading (*n* = 3), mathematical training (*n* = 2), phonological awareness training (*n* = 2), and action video game (*n* = 1). All participant were medication-naïve. The sample sizes in the included studies ranged from 12 to 36 participants (see Table 4 for details). Regarding the assessment procedures employed in the included studies, 4 of them utilized two assessment sessions, consisting of a pre-test and post-test evaluation. On the other hand, 10 studies incorporated three assessment sessions, encompassing a pre-test, post-test, and follow-up evaluation. The follow-up period varied across studies, ranging from 1 week to 6 months. The assessments conducted in the studies can be categorized into three levels: neural, cognitive, and behavioral. Neural assessments, such as evoked potential, utilized physiological measurements to evaluate neural activity. Cognitive assessments, such as the n-back test, involved tasks aimed at measuring the accuracy and/or speed of cognitive performance. Behavioral assessments, such as reading performance, employed objective measures in the form of questionnaire-based evaluations to assess behavioral performance, see Table 8 in Appendix. Table 5 describes and categorizes the properties of intervention programs based on administration (clinician-, parent-, teacher-, or self-administered), objectivity (objective task-based intervention or subjective intervention), and material (computerized, tDCS device, paper and pencil, educational). These properties of assessment tools and intervention programs were utilized in our framework to explore different domains of transfer.

**Table 4:** Characteristics and results of the included studies

First Author, Year	Study design, control condition^1^	Participants: Group; N(DX), A(M ± sd), G (F:M)^2^	tES (site: timing)^3^: I(mA)*ES (cm2)*SN*SD(min)^4^	Additional Intervention^5^ (Timing)^6^: SN*SD (min)^4^	Assessments^7^	Clinical outcome measures and results
Battisti et al. [6]	CO, DB, SS	I: 12 DD, 12.42 ± 2.45, 5:7 C: 12 DD, 14.24 ± 2.68, 5:7	tDCS (PO7/PO8: off): 1*35*5*20	-	HFWT, LFWT, NWT, PBT, RAN, TRT, VNBT, VWMT	Improved non-word reading after intervention and remained until follow-up
Bertoni et al. [8]	CG, DB, SS	I: 10 DD, 22.1 ± 4.65, 7:3 C: 10 DD, 23.6 ± 8.19, 4:6	hf-tRNS (P3-P4: off): 1.5*4*4*12*20	AVG (D): 12*75	NWT, WT, TAT, EEG	AVG training with hf-tRNS enhanced words and pseudo words reading efficiency and visual temporal attention mechanisms
Costanzo et al. [17, 18]	CG, P, DB, SS	I: 9 DD, 13.2 ± 2.6, 5:4 C: 9 DD, 13.6 ± 2.1, 7:2	tDCS (bw P7-TP7/bw P8-TP8: off): 1*25*18*20	CRT (D): 18*20	HFWT, LFWT, NWT, TRT	Improved low frequency word reading accuracy and non-word reading speed immediately and 1 month after treatment
Costanzo et al. [16]	CG, P, DB, SS	I: 13 DD, 13.6 ± 2.4, 8:5 C: 13 DD, 13.9 ± 2.2, 7:6	tDCS (bw P7-TP7/bw P8-TP8: off): 1*25*18*20	CRT (D): 18*20	HFWT, LFWT, NWT, TRT	Improved non-word reading after intervention and remained after two 1- and 6-months follow-up
GholamzadeNikjoo et al. [22]	CG, P, OL	36 DD, I: 11.16 ±.85 C: 11.33 ±.76	tDCS (F3/EC: off): 1.75*25*20*20	-	BDS, BPAQ	Decreased aggression and impulsivity of children in the active group decreased
Heth & Lavidor [26]	CG, P, OL, SS	I: 10 DD, 27.2 ± 7.2, 6:4 C: 9 DD, 24.5 ± 5.2, 4:5	tDCS (V5/ROF: off): 1.5*25*5*20	-	RANL, RANN, SST, TRT	Improved reading speed (1 week after treatment), rapid automatized naming (letter and number) after intervention
Lazzaro et al. [34, 35]	CO, SB, SS	I: 14 DD, 12.54 ± 2.40, 6:8 C: 13 DD, 14.25 ± 2.45, 4:9	tDCS (PO7/PO8: off): 1*25*5*20	-	HFWT, LFWT, NWT, TRT	Improved non-word reading speed in the active group immediately after intervention and remained until one-week
Lazzaro et al. [33]	CG, P, DB, SS	I: 13 DD, 13.6 ± 2.4, 8:5 C: 13 DD, 13.9 ± 2.2, 7:6	tDCS (bw P7-TP7/bw P8-TP8: off): 1*25*18*20	RTT (D): 18*20	WT, NWT	Improved word reading fluency improved after intervention
Looi et al. [39]	CG, P, SS, SB	I: 6 MLD, 114.8 ± 6.6, 1:5 C: 6 MLD, 112.8 ± 8.5, 1:5	tRNS (F3/F4: on and off):.75(.1–500 Hz)*25*9*20	NLT (D): 9*20	CBTT, DST, MALT, NLT	Improved number line accuracy and mathematical abilities after intervention and remained until 4-month follow-up
Mirahadi et al. [43]	CG, P, DB, SS	I: 14 DD, 9.11 ± 1.45, 3:11 C: 14 DD, 9.68 ± 1.07, 6:8	tDCS (bw T3-P3/bw T4-P4: off): 1*35*15*20	PA/GTPC (D&A): 15*60	HFWT, LFWT, NWT, PBT, PDT, RDT	Improved non-word reading and rhyme detection after intervention which remained until follow-up
Oliaee et al. [49]	SG, OL	I: 16 DD, 7.76 ± 1.01, 7:9	tDCS (bw T3-P3/bw T4-P4: off): 1*35*20*20	BWS (D): 20*20	SDT, WISC-V, EEG	Improved dictation and WISC-V scores and normalizing power spectrum density in EEG signals
Rios et al. [57]	SG, OL	I: 12 DD, 12.5 ± 3.18, 3:9	tDCS (bw T3-T5/FP2: off): 2*35*5*30	-	LIT, NWT, ST, TRT, WT	Improved text and non-words reading accuracy
Rufener et al. [60]	CG, P, DB, SS	I: 14 DD, 11.85 ± 2.51, 3:11 C: 15 DD, 11.29 ± 2.37, 4:11	tACS (T7: T8: off): 1(40 Hz)*35*10*20:7000	LST (D&A): 10*40	PPT, TRT, WrT, IGF	Improved phonemic processing skill, writing skill, and gamma frequency in EEG
Srivastav & Chatterjee [68]	SG, OL	I: 13 MLD, 13.85 ± 1.5, 5:8	tDCS (P3/P4: off): 2*25*6*30	CNT (D): 6*30	LDDI	Improved mathematical performance

*Abbreviations*: ^1^*CG* Control Group, *CO* Crossover, *DB* Double Blind, *OL* Open Label, *P* Parallel, *SB* Single Blind, *SG* Single Group, *SS* Sham Stimulation

^2^*A(M ± SD)* Age(Mean** ± **Standard Deviation), *C* Control group, *DD* Developmental Dyslexia, *F* Female, *I* Intervention Group, *M* Male, *MLD* Mathematical Learning Disorder

^3^*bw* between, *EC* extracephalic, *FP* frontoparietal, *Fr* Frontal, *hf-tRNS* high frequency tRNS, *off* offline, *P* Parietal, *PO* parieto-occipita, *ROF* Right Orbito-Frontal, *tACS* transcranial alternating current stimulation, *tDCS* transcranial direct current stimulation, *T* Temporal, *TP* Temporoparietal, *tRNS* transcranial random noise stimulation, *V* Visual

^4^*Cm2* Square Centimeter, *ES* Electrode Size, *Hz* Hertz, *I* Intensity, *mA* Milliampere, *min* Minute, *SD* Session Duration, *SN* Session Number

^5^*AVG* Action Video Game, *BWS* BrainWare Safari, *CNT* Conventional Numeracy Training, *CRT* Cognitive Reading Training, *GTPC* Grapheme to Phoneme Correspondence, *LST* Literacy Skills Training, *NLT* Number Line Training, *PA* Phonological Awareness, *RTT* Reading Training Task

^6^*A* After, *B* Before, *D* During

^7^*BDS* Barratt’s Developmental Scale, *BPAQ* Buss and Perry’s Aggression Questionnaire, *CBTT* Corsi Block-Tapping Test, *DST* Digit Span Test, *EEG* Electroencephalogram, *HFWT* High Frequency Word Task, *IGF* Individual Gamma Frequency, *LDDI* Learning Disability Diagnostic Inventory, *LFWT* Low Frequency Word Task, *LIT* Letter Identification Task, *MALT* Mathematics Assessment for Learning and Teaching, *NLT* Number Line Training, *NWT* Non-Word Task, *PBT* Phoneme Blending Task, *PDT* Phoneme Deletion Test, *PPT* Phonemic Processing Test, *RAN* Rapid Automatized Naming, *RDT* Rhyme Detection Test, *SDT* Standard Dictation Test, *SST* Symbol Search Test, *ST* Syllable Task, *TAT* Temporal Attention Task, *TRT* Text Reading Task, *VNBT* Verbal N-Back Test, *VWMT* Visuospatial Working Memory Test, *WISC-V* Wechsler Intelligence Scale for Children, *WrT* Writing Test, *WT* Word Task

**Table 5:** Intervention, assessment and transfer effect in the included studies

**Author (Year)**	**Intervention’s Fields**					**Assessment’s Fields**					**TI**^**6**^
	**F**^**1**^	**I**^**2**^	**E**^**3**^	**L**^**4**^	**D**^**5**^	**F**^**1**^	**I**^**2**^	**E**^**3**^	**L**^**4**^	**D**^**5**^	
Battisti et al. [6]	NM	COD	C	N	-	Ex	SOP	SC	BC	PPF	3.67
Bertoni et al. [8]	NM/AVG	COD/SOC	C	N/B	-	At	SOP/COC	C	BCN	PPF	4.67
Costanzo et al. [17, 18]	NM/RT	COD/SOC	C	N/B	-	R	SOP	S	B	PPF	4.67
Costanzo et al. [16]	NM/RT	COD/SOC	SC	N/B	-	R	SOP	S	B	PPF	4.67
GholamzadeNikjoo et al. [22]	NM	COD	C	N	-	E	SOP	S	B	PP	2.67
Heth & Lavidor [26]	NM	COD	C	N	-	E	SOP	SC	B	PPF	2.67
Lazzaro et al. [34, 35]	NM	COD	C	N	-	E	SOP	S	B	PPF	3.67
Lazzaro et al. [33]	NM/RT	COD/SOC	SC	N/B	-	R	SOP	S	B	PPF	4.67
Looi et al. [39]	NM/MT	COD/SOC	SC	N/B	-	Ma	SOP/SOC	S	B	PPF	3.67
Mirahadi et al. [43]	NM/PAT	COD/SOP	C	N/B	-	PA	SOP	S	B	PPF	4.67
Oliaee et al. [49]	NM/CT	COD/SOC	C	N/C	-	CT	SOP/COC	SC	BN	PP	3.67
Rios et al. [57]	NM	COD	C	N	-	E	SOC	S	B	PP	2.67
Rufener et al. [60]	NM/LST	COD/SOP	C	N/B	-	LS	SOP/COC	SC	BN	PPF	4.67
Srivastav & Chatterjee [68]	NM/MT	COD/SOP	SC	N/B	-	Ma	SOP	S	B	PP	3.67

*Abbreviations*: ^1^*F *function, *At* Attention, *AVG* Action Video Game, *CT* Cognitive Training, *Ex* Executive Function, *LS* Literacy Skills, *LST* Literacy Skills Training, *Ma* Mathematical, *MT* Mathematical Training, *NM* Neuro Modulation, *PA* Phonological Awareness, *PAT* Phonological Awareness Training, *R* Reading, *RT* Reading Training

^2^*I* investigation tools, *C* Clinician, *P* Parent, *S* Self-administered, *T* Teacher, *O* Objective, *S* Subjective, *C* Computerized, *D* Devised-base, *P* Paper and pencil

^3^*E* ecology, *C* Clinic, *S* School

^4^*L* level, *B* Behavioral, *C* Cognitive, *N* Neural

^5^*D* durability, *PP* Pre-test and Post-test, *PPF* Pre-test, post-test and follow-up

^6^Transfer Investigation Index

**Fig. 2:**
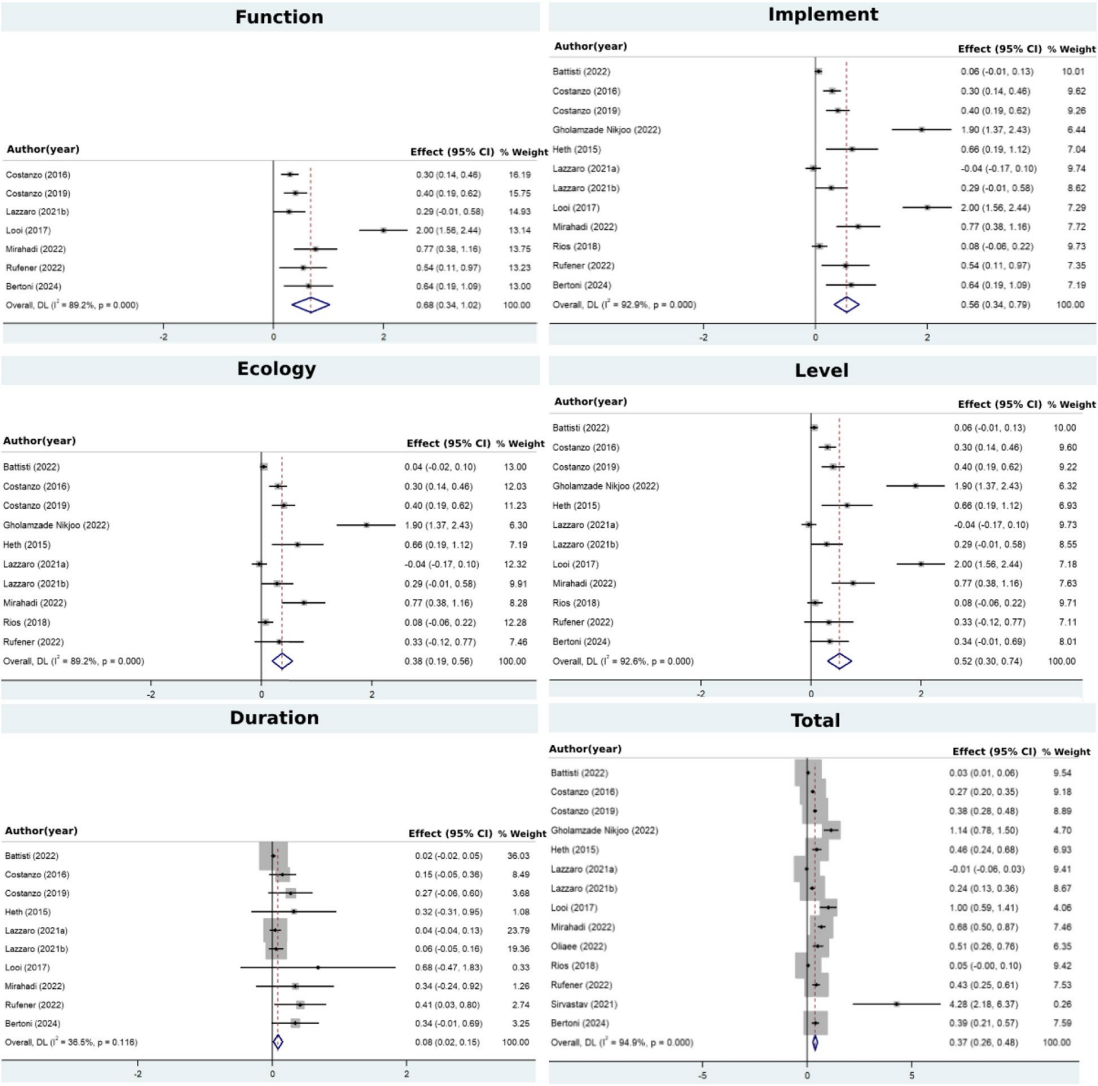
Forest plot illustrating the effect sizes across different domains of the FIELD model. Each horizontal line represents an individual study or domain, with the central marker indicating the estimated effect size and the line reflecting the confidence interval

Table 6 showed the effect sizes and confidence intervals of studies in different domains of FIELD and all domains and overall effect in all studies. For the function, seven studies excluded from analysis because of heterogeneity, Fig. 2. The summary effect size was 0.68 (95% CI: 0.34, 1.02), indicating a moderate positive effect. The homogeneity among the studies (*I*^*2*^ = 89.2%), suggesting that the variability between studies was low, and the overall effect estimate was consistent across the included studies. These findings suggest that the intervention had a substantial and consistent positive impact on the functional transfer, providing moderate evidence for its effectiveness. For the investigation tools, the summary effect size was calculated as 0.56 (95% CI: 0.34, 0.79), signifying a medium positive effect. The moderate homogeneity among the studies (*I*^*2*^ = 92.9%), indicating low variability between studies, and consistency in the overall effect estimate across the included studies. These findings provide robust evidence for the intervention's substantial and consistent positive impact on the measurement-based transfer. For ecological transfer, the summary effect size was calculated to be 0.38 (95% CI: 0.19, 0.58), indicating a low positive effect. All individual study, except one [17, 18] effect sizes demonstrated positive treatment effects on the ecological transfer. The heterogeneity among the studies (*I*^*2*^ = 89.2%), suggesting high level of variability in the included studies.

**Table 6:** The effect sizes of included studies in the FIELD’s domains

**Author (Year)**	Hedge’s g (95% Confidence Interval)					
	F	I	E	L	D	S
Battisti et al. [6]	-	.06 (-.01,.13)	.04 (-.02,.1)	.06 (-.01,.13)	.02 (-.02,.05)	.03 (.01,.06)
Bertoni et al. [8]	.64 (.18, 1.09)	.64 (.18, 1.09)	-	.34 (-.01,.69)	.34 (-.01,.69)	.33 (.21,.57)
Costanzo et al. [17, 18]	.3 (.14,.46)	.3 (.14,.46)	.3 (.14,.46)	.3 (.14,.46)	.15 (-.05,.38)	.27 (.2,.35)
Costanzo et al. [16]	.4 (.19,.62)	.4 (.19,.62)	.4 (.19,.62)	.4 (.19,.62)	.27 (-.06,.6)	.38 (.28,.48)
GholamzadeNikjoo et al. [22]	-	1.9 (1.37, 2.43)	1.9 (1.37, 2.43)	1.9 (1.37, 2.43)	-	1.14 (.78, 1.5)
Heth & Lavidor [26]	-	.66 (.19, 1.12)	.66 (.19, 1.12)	.66 (.19, 1.12)	.32 (-.31,.95)	.46 (.24,.68)
Lazzaro et al. [34, 35]	-	-.04 (-.17,.1)	-.04 (-.17,.1)	-.04 (-.17,.1)	.04 (-.04,.13)	-.01 (-.06,.03)
Lazzaro et al. [33]	.29 (-.01,.58)	.29 (-.01,.58)	.29 (-.01,.58)	.29 (-.01,.58)	.06 (-.05,.16)	.24 (.13,.36)
Looi et al. [39]	2 (1.56, 2.44)	2 (1.56, 2.44)	-	2 (1.56, 2.44)	.68 (-.47, 1.83)	1 (.59, 1.41)
Mirahadi et al. [43]	.77 (.38, 1.16)	.77 (.38, 1.16)	.77 (.38, 1.16)	.77 (.38, 1.16)	.34 (-.24,.92)	.68 (.5,.87)
Oliaee et al. [49]	-	-	-	-	-	.51 (.26,.76)
Rios et al. [57]	-	.08 (-.06,.22)	.08 (-.06,.22)	.08 (-.06,.22)	-	.05 (-.00,.1)
Rufener et al. [60]	.54 (.11,.97)	.54 (.11,.97)	.33 (-.12,.77)	.33 (-.12,.77)	.41 (.03,.8)	.43 (.25,.61)
Srivastav & Chatterjee [68]	-	-	-	-	-	4.28 (2.18, 6.37)
Overall	.68 (.34, 1.02)	.56 (.34,.79)	.38 (.19,.56)	.52 (.3,.74)	.08 (.02,.15)	.37 (.26,.48)
DL (I^2^, *p*- value)	89.2%, <.001	92.9%, <.001	89.2%, <.001	92.6%, <.001	36.5%,.116	94.9%, <.001

*Abbreviations*: *F* function, *I* investigation tools, *E* ecology, *L* level, *D* durability, *DL* DerSimonian-Laird, *I*^*2*^ quantifies the percentage of total variation across studies

For the level of transfer, summary effect size was 0.52 (95% CI: 0.3, 0.74), indicating a significant and large positive effect. All studies except one [34, 35], indicating positive treatment effects on the level transfer. The homogeneity among the studies (*I*^*2*^ = 92.6%), suggesting high heterogeneity between studies. These findings suggest that the intervention had a medium positive impact on the transferability across different level. For the duration, the summary effect size was.08 (95% CI: 0.02, 0.15), indicating a small positive effect. All studies show positive treatment effects on the duration transfer in each study. The homogeneity among the studies (*I*^*2*^ = 36.5%) suggest low homogeneity between studies. These findings suggest that the intervention had a low positive impact on the transferability across time. For the total FIELD domains, the overall effect size was.37 (95% CI: 0.26, 0.48), indicating a low positive transferability effect of intervention. All studies except one [34, 35], indicating positive treatment effects on the total transfer. The homogeneity among the studies (*I*^*2*^ = 94.9%) suggest low heterogeneity between studies. These findings suggest that the intervention had a low positive impact on the transferability across time.

The subgroup analysis unveiled the impact of several potential factors on the transferability of interventions, Table 7. Specifically, the effect size of tES studies with additional interventions surpassed that of studies without additional intervention (0.46 vs. 0.15). Moreover, the dosage of tES was found to exert a significant influence on the effect size. Considering the dyslexia subtypes, the intervention exhibited a more pronounced effectiveness in dyslexia compared to MLD (2.47 vs. 0.32). Lastly, it was observed that the transferability of interventions was more pronounced in the earlier age groups. These findings shed light on the various elements that contribute to the effectiveness and generalizability of interventions across different contexts, providing insights for future research and practice.

**Table 7:** Subgroup analysis of transfer effect size

Potential Factors	Groups	Hedges'g (CI %95)	Number of Studies	Heterogeneity	*P* value
Participants’ Age	< 12	.71 (.48,.95)	5	76.1%	.002
	≥ 12	.21 (.11,.31)	9	94.1%	<.001
SLD Subtype	Dyslexia	.33 (.22,.43)	12	95%	<.001
	Dyscalculia	2.47 (-.72, 5.66)	2	88.9%	.003
tES Dose	< 9000	.28 (.14,.42)	6	93.1%	<.001
	≥ 9000	.45 (.27,.64)	8	94.5%	<.001
Additional Intervention	Yes	.46 (.32,.59)	9	82.5%	<.001
	No	.15 (.05,.26)	5	92.8%	<.001
All studies		.37 (.26,.48)	14	94.9%	<.001

## Discussion

In this study, our objective was to conduct a comprehensive review and analysis on the transferability of NIBS in individuals with SLD based on the FIELD model. Overall, positive transfer effect was found in all domains of FIELD, function, implementdefine, ecology, level, and durability. In the subsequent section, a detailed discussion of our results will provide.

### Functional transfer

The analysis revealed a relatively moderate effect size (0.68) for functional transfer, indicating the effectiveness of tES on other functional aspects. At the function level, the intervention targeted neural excitability, and any changes in the neural readout were considered as functional transfer at the same neural level. In the included studies, three studies registered EEG [49, 60], and the alteration in EEG recording was considered as transfer at the same neural level between different functional areas. Additionally, nine studies [8, 16–18, 34, 39, 43, 49, 60, 68] incorporated additional interventions, where the difference between assessment and intervention function was regarded as functional transfer. These findings highlight the significant impact of tES on functional transfer and its potential to influence various neural processes across different functional domains. With respect to the studies, a large effect size was observed in the case of tRNS over the right and left dlPFC combined with number line training as an additional intervention [39]. Since the functional transfer has been described in this study with an additional intervention, the result cannot solely be attributed to the role of the additional intervention in functional transfer. Earlier studies revealed a transfer of number line training effect to decision making [67] and mathematical skills [23, 67].

### Implementation/measurement transfer

The results demonstrate a medium measurement transfer (effect size = 0.56), highlighting the intervention's influence across various measurements using different materials. Notably, computer-based intervention yielded enhancements in paper-based reading performance, showcasing the versatility and efficacy of the intervention. In children with SLD who have mainly involved with paper and pencil for their academic works, this transfer sounds crucial.

For children with SLD who primarily rely on paper and pencil for their academic tasks, this transfer becomes crucial. Additionally, the independent application of tES intervention regardless of participant involvement unveiled the potential for functional intervention in objective measurements post tES. This underscores the notion that performance improvements are not limited to specific measurements or interventions; rather, any progress in performance can be observed across diverse measurements. These findings underscore the broad applicability and promising implications of the intervention's impact on performance outcomes. Earlier studies reveled improvement in reading and mathematical abilities in SLD after computer-based interventions [5, 64], indicating an investigation tools transfer. In another study, similar reading performance was observed among first-grade students at risk of dyslexia who participated in two programs for fundamental reading skills-one administered by teachers and the other by computer [71].

### Transfer across levels

Given that SLD involve behavioral problems with cognitive and neural impairments, effective interventions targeting each level should ideally transfer their benefits to behavioral levels [45]. In this study, the neural intervention demonstrated a medium effect on other levels (effect size = 0.52), signifying the transferability of the intervention's impact from the neural level to cognitive and behavioral levels in individuals with SLD. This observed transferability serves as a crucial index of intervention effectiveness, highlighting the potential for interventions to yield positive outcomes across various aspects of learning and behavior in those with SLD. The improved behavioral [10] and cognitive [63] performance after tDCS intervention has been described in several met analysis studies in individuals with ADHD.

### Durable transfer

The effect size of transfer in the domain of duration was low (0.08) suggesting the durability of tES intervention in SLD is limited. A meta-analysis study evaluated the follow-up effects of tDCS for major depressive episodes and found significant depression improvement (k = 13, g = −0.81, 95% confidence interval). The longevity of tDCS intervention, coupled with concurrent numerical training on numerical abilities for up to 6 months, renders it a promising and realistic tool for effective intervention [31].

Subgroup analyses were conducted to examine the impact of potential factors on the transfer effect sizes. The findings indicated a positive effect of age, dose, and presence of concurrent intervention on the transferability of interventions, as discussed in the following sections.

### Relevance of age

Subgroup analysis based on age revealed a higher effect size in studies with participants below 12 years old compared to those above 12 (0.71 vs 0.21). These findings align with earlier studies emphasizing the significance of early intervention in addressing SLD as a key factor for response to intervention [56]. As individuals grow older, secondary emotional and behavioral difficulties can complicate interventions [24]. At the neural level, children's brains exhibit greater plasticity compared to adults, which likely contributes to the effectiveness of interventions in younger participants [30]. These results underscore the importance of implementing interventions at an early age to maximize their impact and address SLD challenges more effectively. From an extreme perspective on neurodevelopmental disorders, the critical windows point of view advocates for very early interventions, often implemented before sufficient behavioral symptoms emerge to meet diagnostic thresholds [29].

### Relevance of SLD subtypes

In light of the subtypes of SLD, the intervention demonstrated a greater effectiveness in MLD compared to dyslexia (2.47 vs. 0.33). The specific differences in the effects of tDCS on dyslexia and dyscalculia have not been directly compared in the literature. However, the broader confidence interval observed in dyscalculia, coupled with the limited number of studies, signifies increased uncertainty and a less precise estimate. Earlier studies have delineated a distinct cognitive profile in both dyslexia and dyscalculia, highlighting a phonological deficit in dyslexia and a deficient number module in dyscalculia [32]. These disparities in psychopathology may result in different responses to interventions.

### Relevance of dose

The subgroup analysis for dose of tES revealed a higher efficacy of higher-dose interventions compared to low-dose interventions (0.45 vs. 0.28). This higher impact of higher-dose tES has been previously reported [11, 27, 62, 66]. In the present study, we took into account the total dose, which includes the dose of each single session (equal intensity * duration) multiplied by the number of sessions. Therefore, the observed effects should be attributed to both aspects of the intervention– the intensity and duration of each session and the overall number of sessions conducted. Intensive intervention in SLD has been described as a key factor for response to intervention [56].

### Relevance of concurrent intervention

The subgroup analysis revealed a higher effect size in the studies with additional interventions compared to those without concurrent intervention (0.46 vs. 0.15). The positive impact of concurrent intervention with tDCS has been well-documented in the context of psychotherapy [1, 44, 70], physical therapy [53], cognitive training [12], and pharmacotherapy [73].

In NIBS, anatomical targeting aims to focus on the target cortical areas and enhance performance through the modulation of cortical activity in the target region [51]. Considering the involvement of cortical areas in various tasks across different functional neural networks, introducing a concurrent task adds a functional targeting dimension to the anatomical targeting strategy [9]. By targeting functionally relevant brain areas, tDCS aims to enhance neural activity in specific circuits or networks, leading to potential improvements in targeted functions. The concept of functional targeting in tDCS allows for a more tailored and task-specific approach to neuromodulation. Therefore, the concurrent functional intervention can enhance the effectiveness of tES, as revealed in the present study through improved transferability.

In conclusion, from the review perspective, we highlight the diverse outcomes of tES interventions in relation to different SLD subtypes and contextual factors such as age and dose. The meta-analysis studies provided a quantitative assessment of these effects, revealing a medium effect size for transfer across levels and a low durability effect size, underscoring the need for ongoing reinforcement of tES interventions to sustain benefits over time. Our subgroup analyses further elucidated how age, intervention dose, and concurrent interventions significantly impact the effectiveness of tES, with younger participants benefiting more from early interventions.

This comprehensive review and analysis on the transferability of tES interventions in individuals with SLD based on the FIELD model provides valuable insights into the efficacy and potential impact of these interventions. The results indicate positive transfer effects across various domains of FIELD, including function, investigation tools, ecology, level, and durability. Functional transfer, which demonstrated a relatively large effect size, highlights the effectiveness of tES in influencing other functional aspects by targeting neural excitability. The inclusion of additional interventions further enhances the transferability of the interventions, as observed in various studies.

Moreover, investigation tools transfer revealed the intervention's versatility and efficacy, with computer-based interventions showing enhancements in paper-based reading performance, particularly important for children with SLD who primarily rely on traditional academic tools. Transfer across levels indicates the potential of tES to positively impact behavioral and cognitive aspects in individuals with SLD, emphasizing the importance of interventions that target each level effectively. Additionally, the findings underscore the significance of early intervention and higher intervention doses to maximize effectiveness and better address SLD challenges. The positive impact of concurrent interventions with tES adds further support to the effectiveness of combining interventions to achieve enhanced outcomes. Overall, this study contributes to the growing body of knowledge on NIBS interventions in SLD and highlights their potential as promising tools for intervention and support in individuals with learning difficulties. By understanding the transferability of these interventions across various domains and factors, we can better tailor and optimize interventions to address specific learning challenges effectively, ultimately improving the quality of life and educational outcomes for individuals with SLD. Further research should focus on exploring the long-term effects and potential mechanisms underlying these transfer effects, offering implications for future clinical practice and intervention strategies.

There are certain limitations for this study that should be acknowledged. First, some studies had relatively small sample sizes and heterogeneous designs, which may introduce variability in the effect sizes.. Additionally, the lack of standardized protocols for tES interventions and the varying target areas and methodologies across different studies may introduce potential biases and confounding factors. The inclusion of additional interventions in some studies, while enhancing the transferability of the interventions, may also introduce complexities in attributing the observed effects solely to the tES interventions. Moreover, the specific nature of each additional intervention may vary widely, making it challenging to draw definitive conclusions about their individual contributions to the transfer effects. Finally, it is essential to consider the potential publication bias, as studies reporting significant effects may be more likely to be published than those with non-significant findings. This bias could influence the overall effect size estimates and may not accurately reflect the true magnitude of the transfer effects.

## Data Availability

The data supporting the findings of this study will be available upon reasonable request from the corresponding author.

## Appendix

**Table 8:** Properties of measurements in the included studies

Measurement	Measure(s)	Properties			
		**A** ^**1**^	**O** ^**2**^	**M** ^**3**^	**S** ^**4**^
Barratt’s developmental scale (BDS)	Impulsivity	S	O	P	S
Buss and Perry’s aggression questionnaire, and (BPAQ)	Aggression	S	O	P	S
High Frequency Word Task (HFWT)	Accuracy & speed of reading	S	O	P	S
Learning Disability Diagnostic Inventory (LDDI)	Mathematical performance	S	O	P	S
Letter Identification Task (LIT)	Reading accuracy & speed	S	O	C	S
Low Frequency Word Task (LFWT)	Reading accuracy & speed	S	O	P	S
Mathematics Assessment for Learning and Teaching (MaLT)	Mathematical performance	S	O	P	S
Non-word Task (NWT)	Reading accuracy & speed	S	O	PC	S
Number Line Training (NLT)	Mathematical performance	S	O	C	S
Phoneme Blending Task (PBT)	Phonemic awareness	S	O	P	S
Phoneme Deletion Test (PDT)	Phonemic awareness	S	O	P	S
Phonemic Processing Test (PPT)	Phonemic awareness	S	O	P	S
Reading Task (RT)	Reading accuracy & speed	S	O	PC	S
Rhyme Detection Test (RDT)	Phonemic awareness	S	O	P	S
Standard Dictation Test (SDT)	Writing abilities	S	O	P	S
Syllable Task (ST)	Reading accuracy & speed	S	O	C	S
Word Task (WT)	Accuracy & speed	S	O	PC	S
Writing Test (WrT)	Alphabetic and orthographic strategies	S	O	P	S
Rapid Automatized Naming (RAN)	Reading fluency	S	O	P	S
Corsi Block-Tapping Test (CBTT)	Working memory	S	O	P	S
Digit Span Test (DST)	Working memory	S	O	P	S
Symbol Search Test (SST)	Non-verbal attention	S	O	P	C
Temporal Attention Task (TAT)	Temporal attention	S	O	C	C
Verbal n-Back Test (VnBT)	Working memory	S	O	P	C
Visuospatial Working Memory Test (VWMT)	Working memory	S	O	P	C
Wechsler Intelligence Scale for Children (WISC-V)	Intelligence Quotient	S	O	P	C

*Abbreviation: *^1^*C* Clinician, *P* Parent, *S* Self-administered, *T* Teacher

^2^*O* Objective, *S* Subjective

^3^*C* Computerized, *P* Paper and pencil

^4^*C* Clinic, *H* Home, *S* School
